# Associations between an Invasive Plant (*Taeniatherum caput-medusae*, Medusahead) and Soil Microbial Communities

**DOI:** 10.1371/journal.pone.0163930

**Published:** 2016-09-29

**Authors:** Elise S. Gornish, Noah Fierer, Albert Barberán

**Affiliations:** 1 Department of Plant Sciences, University of California Davis, Davis, California, United States of America; 2 Cooperative Institute for Research in Environmental Sciences, University of Colorado, Boulder, Colorado, United States of America; 3 Department of Ecology and Evolutionary Biology, University of Colorado, Boulder, Colorado, United States of America; University of Oklahoma, UNITED STATES

## Abstract

Understanding plant-microbe relationships can be important for developing management strategies for invasive plants, particularly when these relationships interact with underlying variables, such as habitat type and seedbank density, to mediate control efforts. In a field study located in California, USA, we investigated how soil microbial communities differ across the invasion front of *Taeniatherum caput-medusae* (medusahead), an annual grass that has rapidly invaded most of the western USA. Plots were installed in habitats where medusahead invasion is typically successful (open grassland) and typically not successful (oak woodland). Medusahead was seeded into plots at a range of densities (from 0–50,000 seeds/m^2^) to simulate different levels of invasion. We found that bacterial and fungal soil community composition were significantly different between oak woodland and open grassland habitats. Specifically, ectomycorrhizal fungi were more abundant in oak woodlands while arbuscular mycorrhizal fungi and plant pathogens were more abundant in open grasslands. We did not find a direct effect of medusahead density on soil microbial communities across the simulated invasion front two seasons after medusahead were seeded into plots. Our results suggest that future medusahead management initiatives might consider plant-microbe interactions.

## Introduction

Plant communities are typically composed of a combination of native and non-native species. The majority of these non-native species are benign, demonstrating little to no negative effect on neighboring organisms. However, a small fraction of these non-native plants are characterized as invasive because they are able to profoundly modify local plant and animal communities, nutrient cycling, hydrological regimes and fire frequency [1–2]. Not only do these impacts erode biodiversity and devalue ecosystem services, but they can also enhance further invasion by con- and heterospecific exotics (e.g. [3]).

Soil microbial communities might mediate relationships between invasive plant species and their ecosystem impacts [4–6]. Soil microbial communities, which are typically dominated by fungi and bacteria, can be altered by invasive plants directly through growth facilitation or inhibition near the root zone [7], and indirectly through changes in abiotic conditions (e.g. pH or nutrient availability) that occur in tandem with weed establishment [8]. For example, species-specific effects of non-native grasses on soil nutrients have been shown to subsequently modify soil microbial community composition, biomass, and bacterial:fungal ratios [9]. In addition to being vulnerable to impacts from aboveground plant dynamics, soil microbial communities may also play an important role in mediating the success of plant invasions [4]. For example, extant soil biota have been shown to enhance invasion success of some of the world’s most noxious invasive plants, such as exotic knotweeds (*Fallopia* spp.) [10]. In general, it has been concluded that invasive species may be differentially affected by soil bacterial or fungal pathogens as compared to native plant species [11, 8], but see [12], which could be important for developing reliable control strategies for invasive plants that demonstrate resistance to current management efforts.

The invasive annual winter grass medusahead (*Taeniatherum caput-medusae* [L.] Nevski) has invaded much of the western USA and has been shown to decrease soil carbon stocks, reduce native plant diversity, and enhance fire frequency [13]. A recent meta-analysis of medusahead control outcomes in annual grassland and intermountain regions identified large variance in the effectiveness of conventional approaches for managing medusahead [14], suggesting that underlying variables, such as habitat type and seedbank density, might mediate control efforts. Despite increasing recognition that bacterial and fungal communities can influence plant invasion dynamics, only two published studies have investigated the direct relationship between medusahead and soil microbial communities [15–16]. These studies have conflicting results, suggesting both that the interaction between medusahead and soil microorganisms might or might not enhance its own invasion. Understanding medusahead effects on the soil microbial community is critical for enhancing predictions of invasion effects and for developing effective management strategies.

We investigated how soil microbial communities differ across the invasion front of medusahead in experimental plots in open grassland and oak woodland habitat in the Sierra Foothill region of California, USA. We attempted to understand (1) if medusahead modifies soil microbial communities across the invasion front (simulated by differences in seed density) within systems; and (2) how soil microbial communities differ between areas where medusahead invasion is successful (open grassland habitat) and not successful (oak woodland habitat), and the factors that could be responsible for these differences. We hypothesized that medusahead would modify the soil microbial communities within each habitat. We expected this for two reasons. First, early work on this species [15], as well as more recent work on other invasive annual grasses with similar invasion dynamics to medusahead, have demonstrated linkages between the soil microbes and invasion success [17]. Second, plant-soil interactions are common in the savannah/oak woodlands of California [18], so we would expect strong effects from the extant soil microorganisms. We also hypothesized that soil microbial communities (in particular symbiotic and pathogenic fungi) would differ between areas where medusahead invasion is typically successful (open grasslands) and typically not successful (oak woodlands). In California, invasion of winter annual grasses can be strongly limited within oak canopies [19], possibly due to the different microbial communities associated with oak trees compared to adjacent open grasslands [20–21]. Through shading, litter input, and hydraulic lift, Mediterranean oak trees can also modify a wide variety of soil edaphic factors, such as pH and organic matter concentrations, which directly influence soil microbial communities (e.g. [22]). At present, we do not know what role, if any, soil microbial communities play in mediating the likelihood of successful medusahead invasions.

## Materials and Methods

### Study site and experiment

The study site was located on a research reserve in Yuba County, California (39°14´N, 121°18´W), which experiences a Mediterranean climate of hot, dry summers and cool, wet winters. Permission to perform this experiment was granted by the owner of the property, the University of California Division of Agriculture and Natural Resources. The field study did not involve endangered or protected species.

Mean annual precipitation is 75cm and mean annual temperature is 17.8°C. Soils at the site are fine-loamy, mixed, superactive Ultic Haploxeralfs and fine, mixed, superactive Typic Rhodoxeralfs. Soil pH ranges between 5.7 and 6.2. The experiment is located in an annual grassland system that is irregularly interrupted by small patches of winter-deciduous blue oak (*Quercus douglasii*), and evergreen interior live oak (*Q*. *wislizeni*) that provide approximately 40% shade. The area has experienced seasonal low intensity grazing by livestock since the 1960’s.

The experimental set-up is described fully in [19, 23]. Briefly, plots (1m^2^, separated by 2m) were installed in open grassland habitat and paired oak woodland habitat. The two habitats differ in the identity of dominant herbaceous species and the presence of leaf litter [19]. Average soil temperatures and soil moisture in the top 5cm of soil were also lower in oak woodland plots (18.4°C and 1.6%, respectively) compared to open grassland plots (22.2°C and 6.4%, respectively).

The experimental site was mowed, solarized to enhance seedbank germination and then sprayed with glyphosate herbicide to kill existing and newly germinated plants. In September 2013, fully replicated (n = 4) plots were hand seeded with one of five densities of field-collected medusahead (0, 100, 1000, 10000, and 50000 seeds/m^2^), mixed in with 500 grams of medusahead thatch. Immediately following the addition of medusahead seed, 6,000 seeds each of neighboring grass species (annual rye, and Blando brome) and 4,000 seeds of a clover mix were added—for a total of 16,000 neighbor seeds—to maintain a realistic competitive environment. Medusahead tiller density the following season reflected the seeding rate. This treatment was expected to simulate differences in medusahead invasion intensity from low to high infestation [24].

A defoliation treatment was applied to half the plots in April 2014. This treatment was intended to simulate a mowing or grazing regime included in a typical management program. Defoliation was applied when 75% of the medusahead tillers were in the ‘boot’ stage within a plot. All standing biomass in treatment plots was clipped using electronic shears positioned approximately 15cm above the soil surface.

To understand how factors associated with the oak woodland habitat might contribute to medusahead invasion, an additional set of replicated 1m^2^ plots were installed in the open grassland sites. Treatments were deployed in order to simulate environmental factors associated with the oak woodland habitat, and included the presence of shading, the presence of oak litter, and the presence of both shading and litter. Shading was applied via 50% shade cloth suspended over the plots, and litter was applied by collecting 500g of litter from under paired oak canopies and distributing it evenly over treatment plots. Medusahead seeds (at a density of 50000 seeds/m^2^) were then introduced to these plots.

### Soil sampling

In April 2015, we collected surface soil cores (7cm depth), where the majority of fungal and biomass is present [25] in four random locations in each plot. Soil from each plot was mixed together in the field, sieved and placed in a plastic bag. Soil samples were frozen in the field and transported at -20°C to the University of Colorado, Boulder for microbial analyses.

### Molecular analyses

Microbial diversity was assessed using high-throughput sequencing methods to describe the composition of taxonomic marker gene sequences. For bacterial analyses, we sequenced the V4 hypervariable region of the 16S rRNA gene, using the 515-F (GTGCCAGCMGCCGCGGTAA) and 806-R (GGACTACHVGGGTWTCTAAT) primer pair [26]. For the fungal analyses, we sequenced the first internal transcribed spacer (ITS1) region of the rRNA operon, using the ITS1-F (CTTGGTCATTTAGAGGAAGTAA) and ITS2 (GCTGCGTTCTTCATCGATGC) primer pair [27]. The primers included Illumina adapters and an error-correcting 12-bp barcode unique to each sample. PCR products were quantified using the PicoGreen dsDNA assay, and pooled together in equimolar concentrations for sequencing on an Illumina MiSeq instrument. All sequencing runs were conducted at the University of Colorado Next Generation Sequencing Facility.

Reads were demultiplexed using a custom Python script (https://github.com/leffj/helper-code-for-uparse), with quality filtering and phylotype clustering conducted using UPARSE [28]. For quality filtering, we used a maxee value of 0.5 (that is, a maximum of 0.5 nucleotides incorrectly assigned in every sequence). Singleton sequences were removed prior to phylotype clustering. Quality filtered sequence reads were then mapped to phylotypes at the 97% similarity threshold. Phylotype taxonomy was assigned using the Ribosomal Database Project (RDP) classifier with a confidence threshold of 0.5 [29] trained on the 16S rRNA Greengenes database [30] or the ITS rRNA UNITE database [31], for bacteria and fungi respectively. Sequences representing any phylotypes unclassified at the domain level or classified as mitochondria, chloroplasts, archaea or protists were removed. Subsequently, we removed potential contaminants (i.e. phylotypes with abundances greater than 1% in the blanks and no-template controls [32]), and we normalized the sequence counts using a cumulative-sum scaling approach [33]. We used FUNGuild to identify fungi functional guilds [34]. Soil sample information, phylotype abundance tables, and bacterial and fungal representative sequences are publicly available in FigShare (10.6084/m9.figshare.3113125).

### Statistical analyses

Soil microbial community similarity patterns were represented by non-metric multidimensional scaling (NMDS) using the Bray-Curtis distance metric. We used nested analysis of variance (ANOVA) and permutational multivariate analysis of variance (PERMANOVA) based on 1,000 permutations [35] to assess the explanatory power of the different treatments on soil microbial richness and community similarity patterns, respectively. Differences in the proportion of taxa and fungal functional guilds were tested using non-parametric Wilcoxon tests after false discovery rate (FDR) correction [36]. All multivariate statistical analyses were implemented in the R environment (www.r-project.org) using the vegan package (vegan.r-forge.r-project.org).

## Results

**Fig 1:**
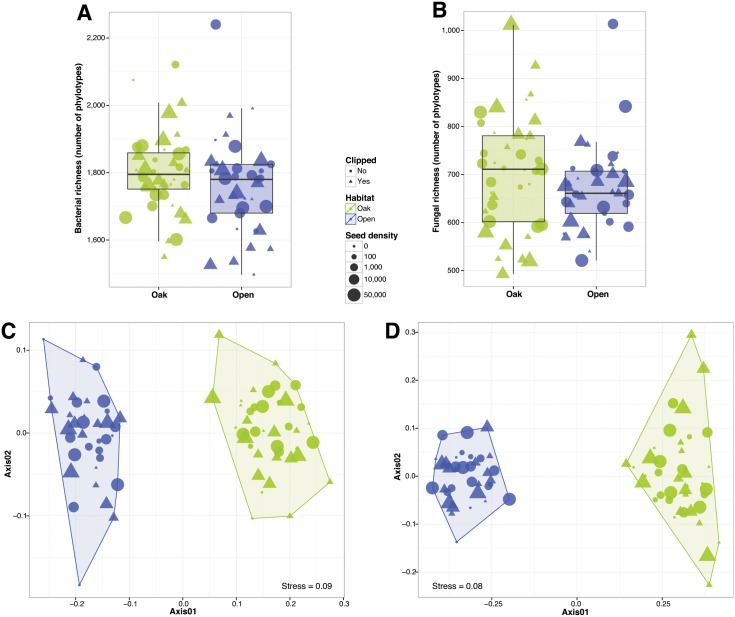
Observed richness (number of different phylotypes per sample) for bacterial and fungal soil communities (A, B). Community similarity patterns for bacterial and fungal soil communities using non-metric multidimensional scaling (C, D).

**Fig 2:**
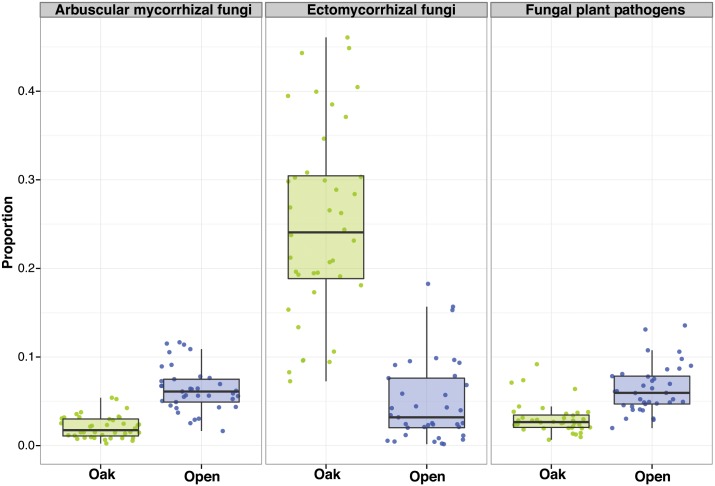
Significant differences (Wilcoxon test P < 0.01 after false discovery rate correction) in the abundance of fungal functional guilds between oak woodland soil samples (green) and open grassland soil samples (blue).

The total number of phylotypes across all soil samples was 5604 and 4349, for bacteria and fungi respectively (S1 Fig). The average number of phylotypes per soil sample was 1781 and 687, for bacteria and fungi respectively. Oak woodland soil samples tended to harbor more bacterial and fungal phylotypes than open grassland samples (although the differences were not statistically significant; ANOVA P > 0.05; Fig 1A and 1B respectively; see S2 Fig for Shannon diversity). Both bacterial and fungal soil community composition were significantly different between open grasslands and oak woodlands (PERMANOVA R^2^ = 0.24, P < 0.001, R^2^ = 0.28, P < 0.001, respectively; Fig 1C and 1D respectively). Bacteria from the proteobacterial classes alpha, beta, gamma and delta, and Acidobacteria subgroup 6 as well as fungi from the Pezizomycetes, Agaricomycetes and Eurotiomycetes classes were more abundant in oak soils than in grassland soils (Wilcoxon test P < 0.01 after FDR; S3 Fig; see S1 Table for results at the genus level). Acidobacteria from the classes Solibacteres and Acidobacteriia, Spartobacteria, Planctomycetia and Phycisphaerae, and fungi from the classes Dothideomycetes, Sordariomycetes and Glomeromycetes were more abundant in open grassland soils (Wilcoxon test P < 0.01 after FDR; S3 Fig; see S1 Table for results at the genus level). As expected from the contrasted fungal taxonomic composition, oak woodlands were different from open grasslands based on the overall abundance of fungal functional guilds (S4 Fig). Ectomycorrhizal fungi (in particular, species from the *Tuber* and *Tomentella* genera) were significantly more abundant in oak woodlands while arbuscular mycorrhizal fungi and plant pathogens (in particular, *Drechslera* and *Fusarium* species) were more abundant in open grasslands (Wilcoxon test P < 0.01 after FDR; Fig 2).

**Fig 3:**
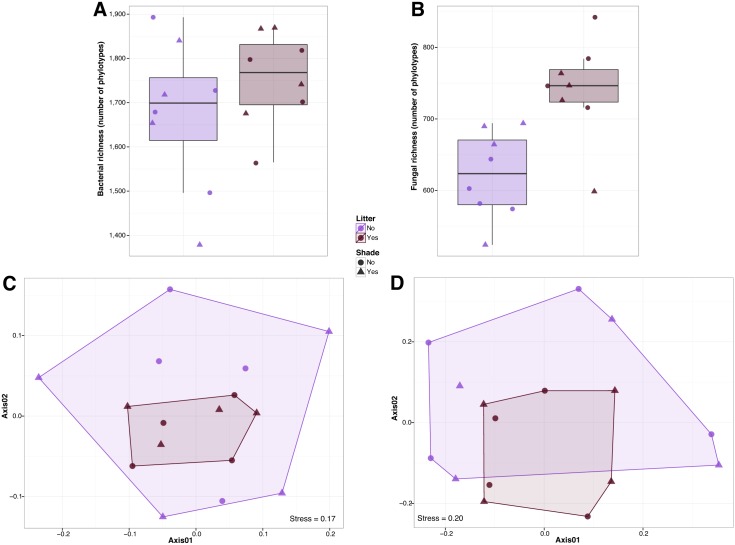
Observed richness (number of different phylotypes per sample) for bacterial and fungal soil communities (A, B). Community similarity patterns for bacterial and fungal soil communities using non-metric multidimensional scaling (C, D).

We found no significant effects of seed density treatments or clipping treatments on soil microbial richness (ANOVA P > 0.05 for both bacteria and fungi; Fig 1A and 1B) or microbial community composition (PERMANOVA P > 0.05 for both bacteria and fungi; Fig 1C and 1D) within each habitat type. Likewise, for those plots where we simulated the oak environment, we observed no significant effects of the litter or shade treatments on the richness of soil bacterial communities (Wilcoxon test P > 0.05; Fig 3A) or bacterial community composition (PERMANOVA P > 0.05; Fig 3C). For fungi, we detected weak significant effects of litter on richness (Wilcoxon test P < 0.01; Fig 3B) and on fungal community composition (PERMANOVA R^2^ = 0.09 P < 0.01; Fig 3D).

## Discussion

Soil biota has been implicated in the facilitation of invasive plant dominance [5, 37]. However, not all invasive species support plant-soil microbe feedbacks as a driver of invasion [38–39]. We attempted to identify how relationships between the weedy annual grass medusahead and soil microorganisms might mediate invasion success. Unexpectedly, we did not find evidence for medusahead density effects on the soil microbial communities across a simulated invasion front in either habitat. This supports other studies that have documented instances where soil communities are unresponsive to the presence of invasive weeds in arid grasslands [40] and in other systems [41]. However, several aspects of our experiment could hinder our ability to capture an existing relationship between medusahead seed density and soil microbial communities. First, we assessed the relationship between medusahead seed density and bulk soil microbial communities and not rhizosphere communities. Rhizosphere microbial communities are different from bulk soil communities [42], so the potential effects of medusahead invasion intensity on microbial communities may be observable at smaller spatial scales in the rhizosphere (but see [43]). Second, although this study was conducted across two growing seasons, there may have been insufficient time for soil microbial communities to respond to different seed densities of medusahead [44]. Although microbial communities have been shown to respond to changes in aboveground plant communities in as little as a month [45], these communities could be especially slow to respond to the presence of weeds in environments where soil edaphic factors are slow to change in response to invasion. Moreover, in California grasslands, soil appears to be particularly buffered from aboveground changes [40]. Third, extracellular microbial DNA and DNA from dead cells can persist in soils for years and thus, obscure DNA-based present estimates of soil microbial composition [46]. Finally, as this study only assessed the composition of the microbial communities, we cannot eliminate the possibility that medusahead seed density can influence the activity and function of belowground soil communities.

Using reciprocal soil transplant experiments, [47] reported higher medusahead biomass in introduced soil than in native soil, which suggests that medusahead success is partially due to release from native soil pathogens [48–49]. In addition to escaping from soil pathogens, certain plant invasive species have been shown to accumulate local pathogens [11, 49]. Although previous studies have reported that medusahead is sensitive to antagonistic fungi [50–51], we observed important differences in bacterial and fungal community composition between open grassland sites where medusahead is typically found in high densities and oak woodland sites where medusahead is typically found in low densities. Specifically, we found significantly higher abundances of fungal pathogens in open grasslands compared to oak woodland habitats. Environmental conditions in the grassland habitat are likely more ideal for both soil and foliar fugal pathogens, which can be important drivers of above ground plant dynamics (e.g. [52]). Indeed, these pathogens have been documented in grasslands in other studies (e.g. [53]). These results collectively highlight the potential contribution of microbial mechanisms (e.g., via pathogen accumulation) to medusahead dominance in California grasslands. Because the differences in bacterial and fungal communities exist in the absence of medusahead, it is more likely that favorable grassland soil microbial communities facilitate medusahead establishment instead of resulting from the invasion itself.

In addition to negative interactions, a large number of plant species establish symbiotic associations with soil microorganisms (in particular with mycorrhizal fungi and nitrogen-fixing bacteria, [6, 54]). In this experiment we detected a higher proportion of ectomycorrhizal fungi in soil samples from oak woodland habitats. This result is expected as ectomycorrhizal fungi are important to oak trees for acquiring nutrients and for increasing root absorptive area [18, 55]. Our results also show that oak litter (rather than shade) influence overall soil fungal community composition and richness, but not soil bacterial community composition and richness. Although plant litter inputs can change important environmental conditions for soil bacteria such as pH and base cation content [42], soil fungi are key decomposers of plant necromass and depend more directly on leaf litter than bacteria [56]. We also observed a significantly higher proportion of arbuscular mycorrhizal fungi in open grasslands. Given the generally non-specific interactions with arbuscular mycorrhizal fungi, it has been proposed that several invasive plants make use of these fungi to enhance their success [57–60]; but see [61]. Other invasive plants (for example, the garlic mustard *Alliaria petiolata*) inhibit mycorrhizal fungi on which natives depend [62]. Collectively, this work suggests that biocontrol and management initiatives should consider the potentially beneficial plant-microbe interactions rather than just focusing on antagonistic relationships.

The context dependency associated with invasion success and weed management efficiency is well documented for both medusahead as well as other weedy species (e.g. [63]). The presence and abundance of bacterial and fungal groups potentially underlie this context dependency in several instances. Environmental changes, such as exacerbated drought conditions, might modify suitability of oak woodland habitat and perhaps enhance invasibility of previously resistant systems. Therefore, given the complex relationships between aboveground and belowground biota [64], understanding the potential mechanisms mediating the association between invasive plant species and soil microorganisms could provide practical information for developing effective management strategies, as well as insight into the ecology of plant-soil food webs and diversity.

## Supporting Information

S1 Fig Sample-based phylotype accumulation curves for bacterial and fungal soil communities. (EPS)

S2 Fig Shannon diversity for bacterial and fungal soil communities (A, B). Differences between open grassland samples and oak woodland samples were statistically significant (ANOVA P < 0.05).
(EPS)

S3 Fig Significant differences (Wilcoxon test P < 0.01 after false discovery rate correction) in the abundance of bacterial and fungal classes between oak woodland soil samples (green) and open grassland soil samples (blue). (EPS)

S4 Fig Abundance of fungal functional guilds between oak woodland soil samples (green) and open grassland soil samples (blue). Note that the y axis is squared.
(PDF)

S1 Table Significant differences (Wilcoxon test P < 0.01 after false discovery rate correction) in the abundance of bacterial and fungal genera between oak woodland soil samples and open grassland soil samples. (DOCX)
