# Supplementary figures and images for: Associations between an Invasive Plant (Taeniatherum caput-medusae, Medusahead) and Soil Microbial Communities
Source: PLoS One. 2016 Sep 29;11(9):e0163930. doi: 10.1371/journal.pone.0163930 (PMC5042559; doi:10.1371/journal.pone.0163930)

**A**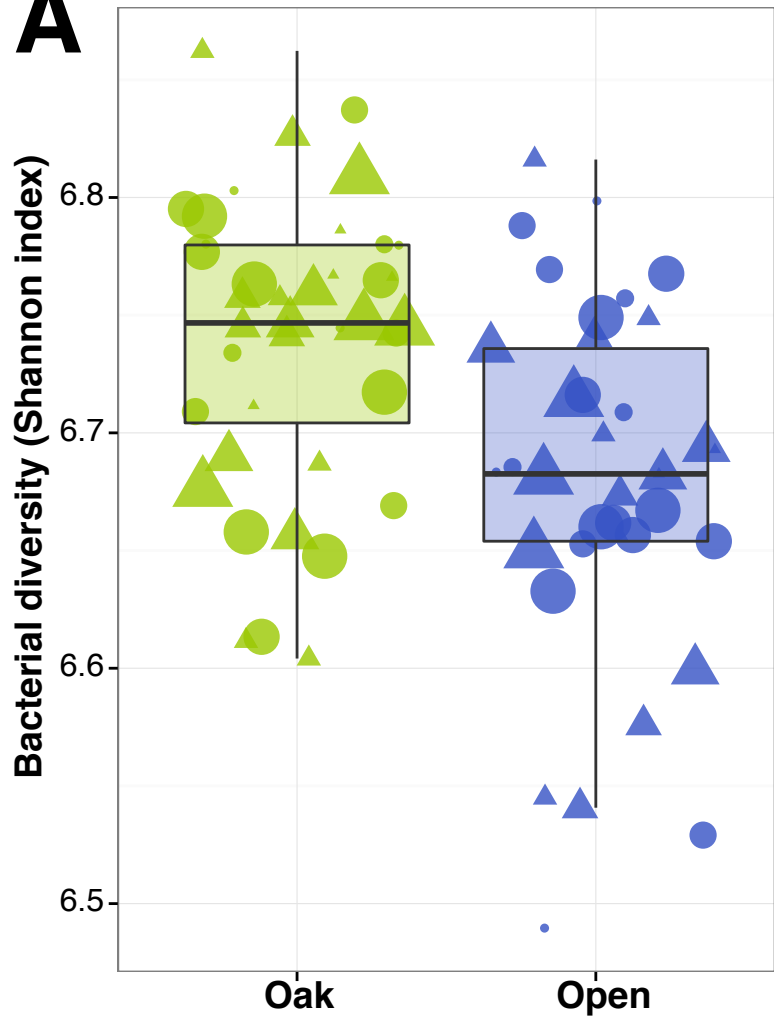**B**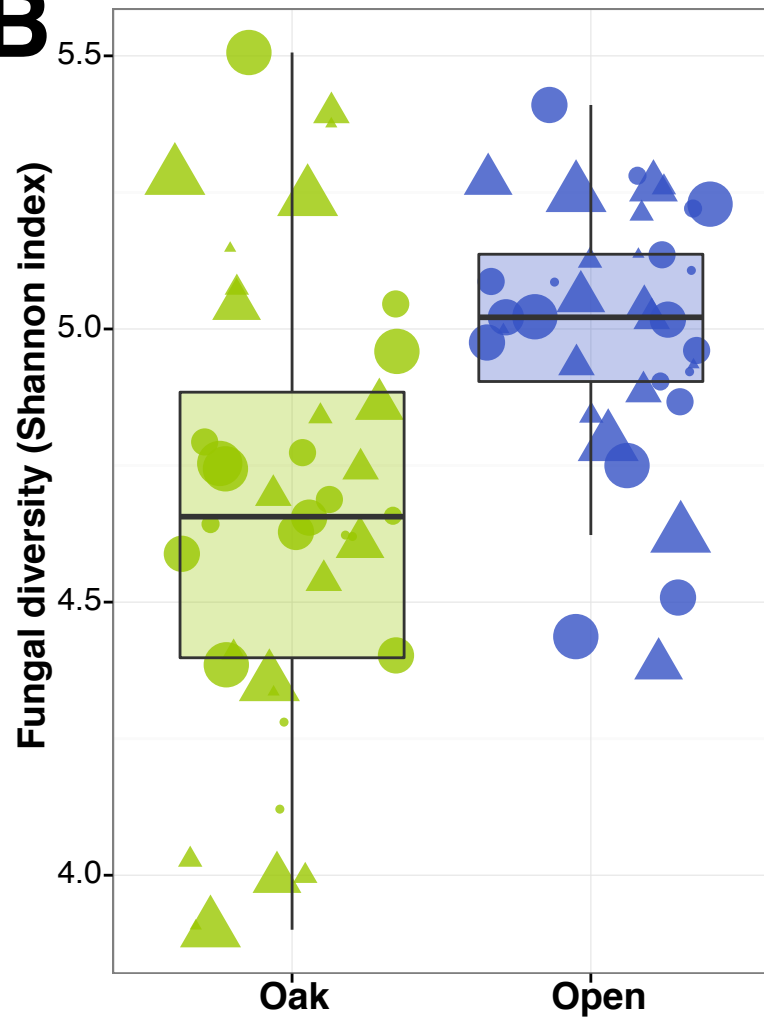

Supplement: S4 Fig — Note that the y axis is squared. (PDF) [file pone.0163930.s004.pdf]
