# Supplementary material for: Associations between an Invasive Plant (Taeniatherum caput-medusae, Medusahead) and Soil Microbial Communities
Source: PLoS One. 2016 Sep 29;11(9):e0163930. doi: 10.1371/journal.pone.0163930 (PMC5042559; doi:10.1371/journal.pone.0163930)
Supplement: S1 Table — (DOCX) [file pone.0163930.s005.docx]

| **Type** | **Genus** | **Oak woodland** | **Open grassland** |
| --- | --- | --- | --- |
| Bacteria | Acidisoma | 5.18E-05 | 0 |
| Bacteria | Actinocatenispora | 3.55E-06 | 6.56E-05 |
| Bacteria | Actinocorallia | 0.000161754 | 1.10E-05 |
| Bacteria | Actinomycetospora | 0.000399872 | 0.000188206 |
| Bacteria | Aeromicrobium | 0.000616278 | 0.000223839 |
| Bacteria | Afifella | 0.00127841 | 0.000129426 |
| Bacteria | Amaricoccus | 6.58E-05 | 3.51E-06 |
| Bacteria | Aminobacter | 0.000793018 | 0.000453912 |
| Bacteria | Arthrobacter | 0.000629942 | 0.000305239 |
| Bacteria | Asteroleplasma | 0.003132313 | 0.0079219 |
| Bacteria | Bacillus | 0.000100242 | 0.000233552 |
| Bacteria | Bosea | 0.001383625 | 0.00085662 |
| Bacteria | Candidatus Koribacter | 0.001589957 | 0.006312042 |
| Bacteria | Candidatus Solibacter | 0.008873589 | 0.015567031 |
| Bacteria | Candidatus Xiphinematobacter | 0.000715669 | 0.00111156 |
| Bacteria | Chitinophaga | 0.000709708 | 0.001444595 |
| Bacteria | Cupriavidus | 6.94E-05 | 0 |
| Bacteria | Cytophaga | 0.000113785 | 0.000608554 |
| Bacteria | Devosia | 0.003089266 | 0.002059491 |
| Bacteria | Erythromicrobium | 0.00011868 | 1.79E-05 |
| Bacteria | Fimbriimonas | 0.00169762 | 0.00238117 |
| Bacteria | Flavisolibacter | 0.008158788 | 0.01637826 |
| Bacteria | Gemmata | 0.009118502 | 0.011948885 |
| Bacteria | Hylemonella | 0.002270361 | 0.003644537 |
| Bacteria | Hyphomicrobium | 0.000652344 | 5.67E-05 |
| Bacteria | Iamia | 0.000592509 | 9.56E-05 |
| Bacteria | Kribbella | 0.001730561 | 0.000887049 |
| Bacteria | Leptolyngbya | 0 | 4.25E-05 |
| Bacteria | Limnohabitans | 0.00539134 | 0.003276918 |
| Bacteria | Luteimonas | 0.000405788 | 0.000146106 |
| Bacteria | Luteolibacter | 0.000887528 | 0.000292132 |
| Bacteria | Mesorhizobium | 0.001419535 | 0.000810219 |
| Bacteria | Methylobacterium | 0.000584277 | 0.000895705 |
| Bacteria | Modestobacter | 0.000482152 | 0.001238739 |
| Bacteria | Nannocystis | 0.000344967 | 0.000168717 |
| Bacteria | Niabella | 0.003297581 | 0.002552388 |
| Bacteria | Niastella | 0.000146284 | 1.89E-05 |
| Bacteria | Nitrosovibrio | 0.000725576 | 0.000125191 |
| Bacteria | Nitrospira | 0.000937845 | 8.10E-05 |
| Bacteria | Nocardia | 0.000204585 | 7.23E-05 |
| Bacteria | Nocardioides | 0.001915181 | 0.00082082 |
| Bacteria | Nonomuraea | 0.000391051 | 0.000122459 |
| Bacteria | Opitutus | 0.005991202 | 0.008179807 |
| Bacteria | Oryzihumus | 3.46E-05 | 0.000185478 |
| Bacteria | Parvibaculum | 7.13E-05 | 9.46E-06 |
| Bacteria | Pedomicrobium | 0.00074436 | 9.78E-05 |
| Bacteria | Pedosphaera | 4.46E-05 | 0.000273423 |
| Bacteria | Phormidium | 1.08E-05 | 0.000442124 |
| Bacteria | Pilimelia | 0.000880488 | 0.000355814 |
| Bacteria | Pirellula | 0.00232937 | 0.003225972 |
| Bacteria | Planctomyces | 0.005655295 | 0.004454444 |
| Bacteria | Polaromonas | 0.002066838 | 0.001490913 |
| Bacteria | Pseudomonas | 0.004720461 | 0.002952186 |
| Bacteria | Pseudonocardia | 0.005250408 | 0.002697026 |
| Bacteria | Pseudoxanthomonas | 0.000333357 | 2.12E-05 |
| Bacteria | Rhodanobacter | 0.000150687 | 2.58E-05 |
| Bacteria | Rhodobacter | 0.000138924 | 1.32E-05 |
| Bacteria | Rhodopila | 0.000102007 | 0.000835611 |
| Bacteria | Rhodoplanes | 0.024147881 | 0.013773826 |
| Bacteria | Sediminibacterium | 0.001558403 | 0.004805921 |
| Bacteria | Segetibacter | 0.00590189 | 0.008851971 |
| Bacteria | Sorangium | 1.58E-05 | 9.21E-05 |
| Bacteria | Sphingomonas | 0.018109974 | 0.010946578 |
| Bacteria | Sphingopyxis | 6.61E-05 | 1.35E-05 |
| Bacteria | Spirosoma | 0.00066076 | 0.001576516 |
| Bacteria | Sporichthya | 0.000260223 | 6.98E-05 |
| Bacteria | Sporocytophaga | 0.000254903 | 0.001081257 |
| Bacteria | Steroidobacter | 0.004153715 | 0.001502732 |
| Bacteria | Streptosporangium | 0.000623202 | 0.000624067 |
| Bacteria | Thermomonas | 0.001452479 | 9.56E-05 |
| Bacteria | Uliginosibacterium | 4.64E-05 | 0.000293076 |
| Bacteria | Virgisporangium | 0.001277844 | 0.000498303 |
| Fungi | Acaulospora | 0.000112203 | 0.001122273 |
| Fungi | Agonimia | 0 | 0.000590421 |
| Fungi | Apodus | 0.02322565 | 0.009192916 |
| Fungi | Arachnopeziza | 0.001940809 | 1.04E-05 |
| Fungi | Arnium | 1.61E-05 | 0.011744784 |
| Fungi | Ascochyta | 0.000198154 | 5.05E-06 |
| Fungi | Bionectria | 0.001809484 | 0.000372359 |
| Fungi | Botrytis | 0.00269665 | 1.99E-05 |
| Fungi | Bullera | 4.11E-05 | 0.000331609 |
| Fungi | Calvatia | 2.90E-05 | 0.000522309 |
| Fungi | Calyptella | 0.002752027 | 2.07E-05 |
| Fungi | Camposporium | 0.0017827 | 0.000362814 |
| Fungi | Celosporium | 0.000308611 | 2.37E-05 |
| Fungi | Ceratobasidium | 0.006668568 | 0.018402794 |
| Fungi | Chaetomium | 0.008044104 | 0.00211121 |
| Fungi | Chaetosphaeronema | 2.84E-05 | 0.000874094 |
| Fungi | Cladophialophora | 0.02372422 | 0.010719984 |
| Fungi | Claroideoglomus | 0.000542808 | 0.001575957 |
| Fungi | Clavaria | 0.005179704 | 0.042100103 |
| Fungi | Collophora | 0.000229285 | 0.022214067 |
| Fungi | Coniochaeta | 0.002189779 | 0.009386917 |
| Fungi | Cordyceps | 0.002322751 | 0.000461836 |
| Fungi | Cosmospora | 0.000452646 | 0.002570403 |
| Fungi | Cristinia | 0.000246365 | 0 |
| Fungi | Cryptococcus | 0.013259355 | 0.017902619 |
| Fungi | Cryptosporiopsis | 0.000472198 | 7.00E-05 |
| Fungi | Cylindrium | 0.00857061 | 0.000895581 |
| Fungi | Cylindrosympodium | 0.001467198 | 0.000201098 |
| Fungi | Cyphellophora | 0.000932044 | 3.65E-05 |
| Fungi | Cystodendron | 3.91E-06 | 0.000626638 |
| Fungi | Didymella | 0.000245627 | 0.002575308 |
| Fungi | Dinemasporium | 0.002880607 | 0.000292061 |
| Fungi | Drechslera | 0.003716968 | 0.026252873 |
| Fungi | Emericella | 0.001406625 | 0.000397227 |
| Fungi | Endoconidioma | 0.00129976 | 0.000492014 |
| Fungi | Entoloma | 0.005319859 | 0.014851583 |
| Fungi | Eocronartium | 1.15E-05 | 0.000237132 |
| Fungi | Epicoccum | 0.003838529 | 0.010957388 |
| Fungi | Eucasphaeria | 0.001014155 | 2.57E-05 |
| Fungi | Eupenicillium | 0.001329885 | 3.33E-05 |
| Fungi | Exophiala | 0.021232324 | 0.013808775 |
| Fungi | Funneliformis | 0.002609304 | 0.008131384 |
| Fungi | Fusarium | 0.004006292 | 0.026937594 |
| Fungi | Genea | 0.034388181 | 6.65E-05 |
| Fungi | Geniculospora | 0.000414558 | 1.02E-05 |
| Fungi | Geopora | 0.013851928 | 1.90E-05 |
| Fungi | Glomus | 0.008191347 | 0.026049312 |
| Fungi | Gymnopus | 0.008360786 | 2.13E-05 |
| Fungi | Herpotrichia | 0.000123158 | 0 |
| Fungi | Hirsutella | 0.000147258 | 0 |
| Fungi | Humicola | 0.004616596 | 0.000510387 |
| Fungi | Hymenoscyphus | 0.002427637 | 0.008383351 |
| Fungi | Hypomyces | 0.000163858 | 0 |
| Fungi | Ijuhya | 0.000187949 | 1.48E-05 |
| Fungi | Inocybe | 0.052501993 | 0.001679186 |
| Fungi | Kurtzmanomyces | 0 | 0.000166417 |
| Fungi | Laetisaria | 1.06E-05 | 0.003753253 |
| Fungi | Lasiosphaeria | 0.001524295 | 0.000206582 |
| Fungi | Lecanicillium | 0.0018394 | 0.000271823 |
| Fungi | Leohumicola | 0.00383021 | 0.001830374 |
| Fungi | Leptosphaeria | 0.003663957 | 0.000516838 |
| Fungi | Limonomyces | 0.002398788 | 0.003517126 |
| Fungi | Lophiostoma | 0.002272861 | 0.020935731 |
| Fungi | Macrophomina | 8.32E-06 | 0.000114734 |
| Fungi | Marchandiomyces | 0.000660728 | 0.007167487 |
| Fungi | Mastigobasidium | 2.40E-05 | 0.002456117 |
| Fungi | Melanogaster | 0.001452338 | 0 |
| Fungi | Meliniomyces | 0.008568062 | 0.000389619 |
| Fungi | Microdochium | 0.003782441 | 8.16E-05 |
| Fungi | Mycena | 0.002280215 | 5.65E-06 |
| Fungi | Mycoleptodiscus | 0.001380861 | 2.86E-05 |
| Fungi | Myrmecridium | 0.000202843 | 0 |
| Fungi | Myxocephala | 7.32E-06 | 0.000459599 |
| Fungi | Nectria | 0.001036258 | 0.000222072 |
| Fungi | Neonectria | 0.006676322 | 0.000771155 |
| Fungi | Neophaeosphaeria | 0.001397882 | 0 |
| Fungi | Neosartorya | 0.00031513 | 0 |
| Fungi | Neurospora | 4.18E-05 | 0.000703026 |
| Fungi | Ochroconis | 0.00591994 | 0.000150996 |
| Fungi | Oliveonia | 0.001386504 | 0 |
| Fungi | Ophiosphaerella | 0.000750925 | 0.000308286 |
| Fungi | Otidea | 0.000260416 | 0 |
| Fungi | Paecilomyces | 0.002062801 | 0.001212925 |
| Fungi | Panaeolus | 4.97E-05 | 0.015097766 |
| Fungi | Paraconiothyrium | 0.000501125 | 4.33E-05 |
| Fungi | Penicillium | 0.017621019 | 0.0036924 |
| Fungi | Periconia | 0.003360679 | 0.018775961 |
| Fungi | Pestalotiopsis | 0.000268469 | 0.000116996 |
| Fungi | Peziza | 0.012548654 | 0.000377123 |
| Fungi | Phaeococcomyces | 0.000127112 | 0.001814097 |
| Fungi | Phialophora | 0.0006042 | 6.77E-05 |
| Fungi | Phlogicylindrium | 0.000672375 | 5.63E-05 |
| Fungi | Pilidium | 0.000767103 | 6.16E-05 |
| Fungi | Pleiochaeta | 0.000424221 | 4.91E-05 |
| Fungi | Pluteus | 0.000249284 | 0 |
| Fungi | Podospora | 0.015246351 | 0.060480843 |
| Fungi | Protomyces | 0.000284982 | 0.001036533 |
| Fungi | Psathyrella | 0.000122254 | 5.74E-06 |
| Fungi | Pyrenochaeta | 0.004581929 | 0.001737521 |
| Fungi | Rachicladosporium | 5.77E-05 | 0.001144475 |
| Fungi | Rhinocladiella | 0.001069878 | 4.00E-06 |
| Fungi | Rhizoctonia | 0.000389249 | 0.002287365 |
| Fungi | Rhizophagus | 0.001282438 | 0.006966772 |
| Fungi | Rhizopogon | 0.00822366 | 3.36E-05 |
| Fungi | Rhodotorula | 0.003944017 | 0.000889153 |
| Fungi | Rhynchosporium | 9.66E-05 | 1.02E-05 |
| Fungi | Schizothecium | 0.001310083 | 0.001341778 |
| Fungi | Sclerostagonospora | 0.026867815 | 0.04721595 |
| Fungi | Sclerotinia | 1.27E-05 | 0.002125712 |
| Fungi | Sebacina | 0.006750641 | 0.000536007 |
| Fungi | Sistotrema | 0.000357574 | 0.00505975 |
| Fungi | Spiromyces | 0 | 6.55E-05 |
| Fungi | Sporobolomyces | 0.000761552 | 3.07E-05 |
| Fungi | Stilbella | 0.000402736 | 0.000111665 |
| Fungi | Tarzetta | 0.000797678 | 0 |
| Fungi | Tetrachaetum | 1.36E-05 | 0.000593093 |
| Fungi | Tomentella | 0.081428717 | 0.008249733 |
| Fungi | Trichoderma | 0.005632304 | 0.002005426 |
| Fungi | Trichosporon | 0.000185782 | 0 |
| Fungi | Tubaria | 4.13E-06 | 0.003586444 |
| Fungi | Tubeufia | 0.000252151 | 1.31E-05 |
| Fungi | Unguicularia | 0.000775322 | 0.00496177 |
| Fungi | Volutella | 0.000975734 | 4.85E-05 |
| Fungi | Westerdykella | 3.71E-06 | 0.000313223 |
